# Impact of concurrent epidemics of dengue, chikungunya, zika, and COVID-19

**DOI:** 10.1590/0037-8682-0837-2020

**Published:** 2021-02-26

**Authors:** Creuza Rachel Vicente, Theresa Cristina Cardoso da Silva, Larissa Dell’Antonio Pereira, Angelica E. Miranda

**Affiliations:** 1 Universidade Federal do Espírito Santo, Departamento de Medicina Social, Vitória, ES, Brasil.; 2 Universidade Federal do Espírito Santo, Programa de Pós-Graduação em Doenças Infecciosas, Vitória, ES, Brasil.; 3 Secretaria de Estado da Saúde do Espírito Santo, Vigilância em Saúde, Vitória, ES, Brasil.; 4 Universidade Federal do Espírito Santo, Programa de Pós-Graduação em Saúde Coletiva, Vitória, ES, Brasil.

**Keywords:** Arbovirus infections, Coronavirus infections, Epidemics, Health services, Brazil

## Abstract

**INTRODUCTION:**

This study evaluated the epidemiological implications of arbovirus infections and coronavirus disease (COVID-19) co-occurrences in Espírito Santo, Brazil.

**METHODS:**

This ecological study of dengue, chikungunya, zika, and COVID-19 was performed from January 1 to July 31, 2020.

**RESULTS:**

Espírito Santo registered 44,614, 8,092, 3,138, and 91,483 cases of dengue, chikungunya, zika, and COVID-19, respectively (January-July, 2020). In the 27 and four municipalities with a high incidence of dengue and chikungunya, respectively, the incidence of COVID-19 was 647.0-3,721.7 and 1,787.2-3,403.0 cases per 100,000 inhabitants, respectively.

**CONCLUSIONS:**

Espírito Santo experienced an overlap of epidemics, especially in urban areas.

Viral infections transmitted by *Aedes aegypti*, such as dengue, chikungunya, and zika, are significant public health concerns in Brazil and other countries with the simultaneous occurrence of these diseases. The coronavirus disease (COVID-19) pandemic caused by severe acute respiratory syndrome coronavirus 2 (SARS-CoV-2) has imposed additional challenges in territories with overlapping epidemics, increasing the demand for healthcare services^1^ ^-^ ^4^. This scenario was observed in areas where the COVID-19 pandemic began during the seasonal transmission of dengue in Latin America^1^ ^,^ ^3^ ^,^ ^5^ ^,^ ^6^ and Asia^7^. Quarantine and lockdowns adopted in response to the COVID-19 pandemic may have contributed to arbovirus outbreaks as the population maintained close and long contact with mosquito breeding sites in and around their homes^8^.

Dengue, chikungunya, zika, and COVID-19 may present with similar clinical manifestations and laboratory features in the early stages of the diseases, such as an acute undifferentiated febrile illnesses^1^ ^-^ ^3^ ^,^ ^7^. Therefore, diagnosis is challenging even when confirmatory tests are available in cases of COVID-19 presenting with false-positive results for dengue infection^4^ ^,^ ^9^ ^,^ ^10^. Consequently, misdiagnosis may delay appropriate care and management of the disease, such as isolation of patients with COVID-19 and hydration in those with dengue, resulting in increased disease spread and worst clinical outcomes^8^. Moreover, this epidemiological scenario represents a risk of coinfections, as observed in patients diagnosed with dengue and COVID-19^11^.

In Espírito Santo State, Brazil, an area with co-circulation of dengue, chikungunya, and zika viruses, the first case of COVID-19 was reported in February 2020 during the seasonal period of arboviral transmissions. The local government adopted many actions during the COVID-19 pandemic such as suspension of activities in educational, commercial, financial, and alimentary sectors. The present study evaluated the concurrent occurrence of these arbovirus infections and COVID-19 in the state and the possible implications of this epidemiological scenario.

An ecological study was performed based on the number of probable cases of dengue, chikungunya, and zika and confirmed cases of COVID-19 reported to the Health Department of the Espírito Santo State from January 1, 2020 to July 31, 2020 and accessed on September 1, 2020.

The Espírito Santo State is located in the southeast region of Brazil and has 78 municipalities in a 46.089,390 km^2^ land area. The state’s population is estimated to be approximately 4,018,650 individuals. Its climate is tropical humid, with an annual precipitation of >1,400 mm and an average temperature of 23°C^12^.

Publicly available official data on the reports of dengue, chikungunya, and zika were accessed (https://mosquito.saude.es.gov.br/planilhasegraficos) along with those of COVID-19 (https://coronavirus.es.gov.br/painel-covid-19-es). Data on the municipalities’ population were obtained from the same source as that for the arbovirus data.

The definitions of probable and confirmed cases of the diseases used by the Secretary of Health in Espírito Santo State follow the Brazilian Ministry of Health criteria^13^ ^,^ ^14^. A case of dengue is characterized by fever, with a duration of 2-7 days, and two or more manifestations, such as nausea or vomiting, exanthema, myalgia or arthralgia, headache with retro-orbital pain, petechia, positive tourniquet test, and leucopenia^13^. A case of chikungunya is defined as a sudden onset fever (>38.5°C) and arthralgia or severe acute onset arthritis not explained by other conditions. The duration of this acute phase of chikungunya fever is approximately 7 days^13^. A case of zika presents with a pruritic maculopapular rash accompanied by two or more signs and symptoms, such as low fever, conjunctival hyperemia without secretion or itching, polyarthralgia, and periarticular edema for a duration of 4-7 days^13^. Probable cases of dengue, chikungunya, and zika were classified according to their clinicoepidemiological criteria. A confirmed case of dengue, chikungunya, and zika was defined as positivity for viral isolation based on non-structural glycoprotein-1 (enzyme-linked immunosorbent assay [ELISA] or rapid test), quantitative reverse transcription polymerase chain reaction (RT-qPCR), or ELISA IgM; RT-qPCR, ELISA IgM, or IgG (ELISA or hemagglutination test); and RT-qPCR or ELISA IgM, respectively^13^. A case of COVID-19 presents with at least two of the following signs and symptoms: fever associated with sore throat, headache, cough, or runny nose^14^. A case of COVID-19 can be confirmed using the clinical criteria (flu syndrome or severe acute respiratory syndrome with acute anosmia and ageusia), clinicoepidemiological criteria (flu syndrome or severe acute respiratory syndrome and close contact with a confirmed case of COVID-19 in the last 14 days before the onset of symptoms), clinical criteria with specific alterations on chest computed tomography, or laboratory criteria based on a positive RT-qPCR result, immunology with IgM/IgA/IgG (ELISA, immunochromatography, electrochemiluminescence immunoassay), or antigen detection (immunochromatography)^14^.

Descriptive analysis of simple frequency was conducted considering the weekly reports between the epidemiological weeks 1 to 31 (January 1, 2020 to July 31, 2020). The incidences of dengue, chikungunya, zika, and COVID-19 per 100,000 inhabitants were calculated for all municipalities for the entire study period. Maps were produced using QGIS 3.14.15 and shapefile (https://geobases.es.gov.br/downloads). The analyses were performed using Microsoft Excel^®^ 2013 (© 2012 Microsoft Corporation). The cumulative incidence of arbovirus infections was presented in three levels-low (<100 cases per 100,000 inhabitants), medium (100-300 cases per 100,000 inhabitants), and high (>300 cases per 100,000 inhabitants), according to the Health Department of Espírito Santo State criteria. The cumulative incidence of dengue was presented similarly according to the Brazilian Ministry of Health criteria^15^. This study was conducted using online open-access data; therefore, institutional ethics committee approval does not apply.

From January 1, 2020 to July 31, 2020, the Espírito Santo State Surveillance Department registered a high number of cases and incidences of dengue, chikungunya, and zika (Table 1). The incidence per 100,000 inhabitants, considering probable cases, surpassed 300 cases in 27 and four municipalities for dengue and chikungunya, respectively, and was >100 in four municipalities for zika (Figure 1, Supplementary Table 1). The peak incidence of these arbovirus infections was reported between February and April 2020 (Figure 2). The registered deaths due to dengue and chikungunya are shown in Table 1.

**FIGURE 1:**
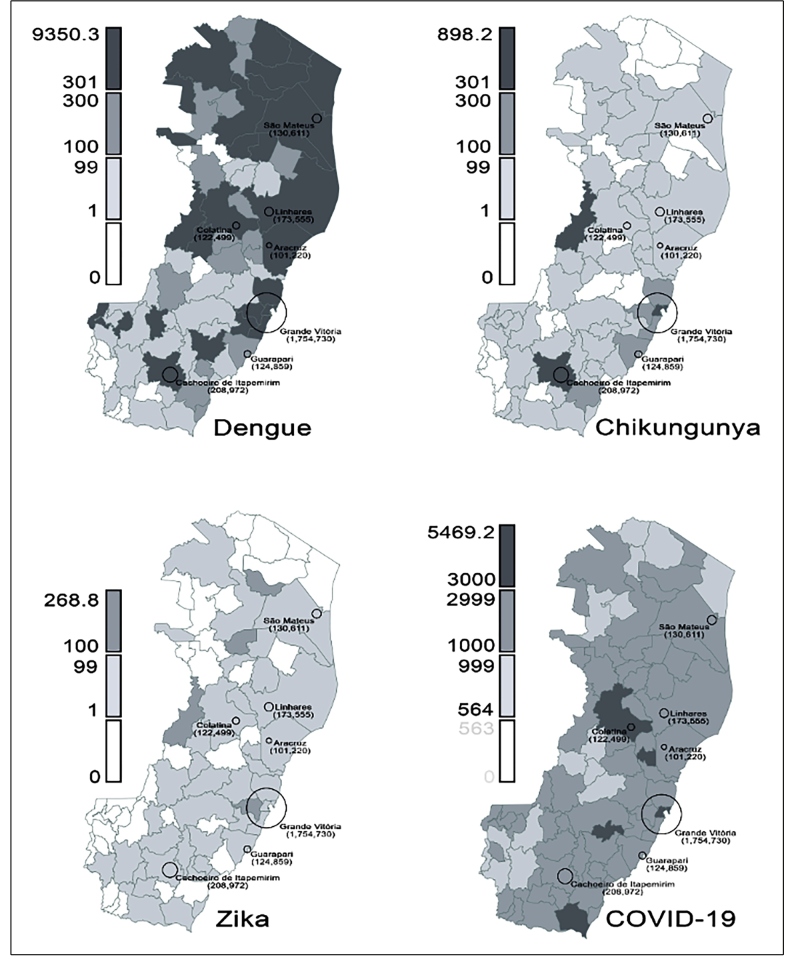
Incidence of dengue, chikungunya, zika, and COVID-19 cases per 100,000 inhabitants in the municipalities of Espírito Santo State from January to July 2020. Circles correspond to the population size in municipalities with >100,000 inhabitants. **COVID-19:** coronavirus disease.

**TABLE 1:** Characteristics of dengue, chikungunya, zika and COVID-19 cases in Espírito Santo State, Brazil from January to July 2020.

Reports characteristics	Dengue	Chikungunya	Zika	COVID-19
Probable cases	44,614	8,092	3,138	91,483
Laboratory-confirmed cases	6,814	2,351	650	84,278
Incidence per 100,000 inhabitants	1,110.2	201.4	78.1	2,276.5
Confirmed deaths	14	3	0	2,909

COVID-19: coronavirus disease.

From February 29, 2020 to July 31, 2020, the state registered 91,483 confirmed cases of COVID-19. Laboratory confirmation was obtained in 92.1% cases, while the clinicoepidemiological criteria was used to confirm other cases (Table 1). The incidence of COVID-19 was >3,000 cases per 100,000 inhabitants in five municipalities (Figure 1, Supplementary Table 1). The peak incidence of COVID-19 was reported between May and July 2020 (Figure 2). Health professionals accounted for 14.2% of the affected individuals, accounting for 12,974 cases of COVID-19. Of the registered deaths due to COVID-19, 2,885 deaths were caused by COVID-19, while 24 deaths were related to other causes (Table 1).

**FIGURE 2:**
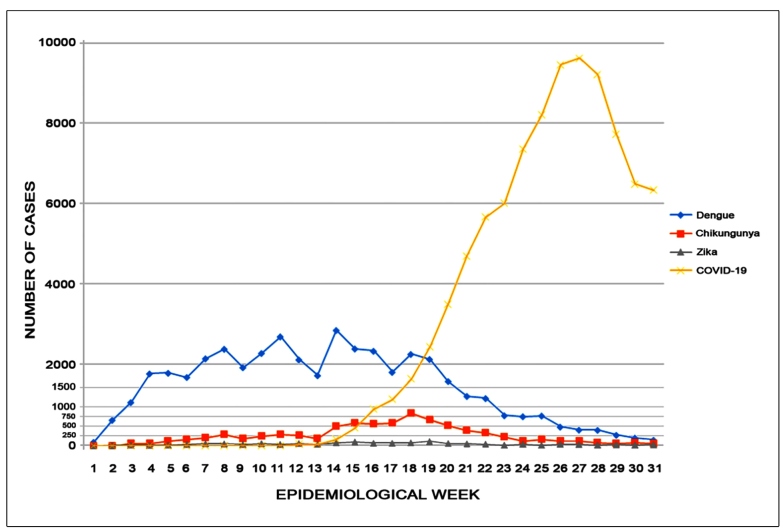
Number of cases of dengue, chikungunya, zika, and COVID-19 per epidemiological week in Espírito Santo State from January to July 2020. Month (epidemiological week): January (1-5), February (5-9), March (10-14), April (14-18), May (18-23), June (23-27), and July (27-31). COVID-19: coronavirus disease.

Four municipalities experienced a high incidence of dengue and chikungunya, with an incidence of >300 cases per 100,000 inhabitants for both diseases. In these municipalities, the incidence of COVID-19 was 1,787.2-3,403.0 cases per 100,000 inhabitants. In the 27 municipalities with a high incidence of dengue, the incidence of COVID-19 was 647.0-3,721.7 cases per 100,000 inhabitants, while in the four municipalities with a high occurrence of chikungunya, the incidence of COVID-19 was 1,787.2-3,403 cases per 100,000 inhabitants. In the four municipalities with an incidence of zika of >100 cases per 100,000 inhabitants, the incidence of COVID-19 was 1,787.2-2,761.7 cases per 100,000 inhabitants **(** Figure 1, Supplementary Table 1).

Espírito Santo State experienced an overlap of epidemics with the introduction of SARS-CoV-2 infection by travelers, followed by its local and community transmission during the peak of dengue, chikungunya, and zika seasonal transmissions. Urban areas were more affected than rural areas, especially in the metropolitan region and municipalities that are regional hubs of economic development. Areas with high human population density favored the transmission of COVID-19 and the arbovirus infections reported in this study, since urban environment is propitious for breeding sites for *A. aegypti* ^8^. This scenario is challenging because of the clinical similarities between the diseases and the limitations related to the confirmatory tests^1^ ^-^ ^4^ ^,^ ^7^ ^,^ ^9^ ^,^ ^10^.

Health systems dealing with concomitant infectious disease epidemics tend to experience challenges in their different service areas, including laboratory, primary healthcare, hospital, and epidemiological surveillance systems. The high incidence of COVID-19 among health professionals in Espírito Santo State, which may affect the capacity of the health system, emphasizes the importance of this challenge. In this state, <30% of dengue, chikungunya, and zika cases were confirmed by laboratory tests. Although laboratory confirmation for arbovirus transmission in endemic countries has a surveillance purpose^4^, the clinical management of arbovirus infections may also require follow-up with laboratory parameters, such as hematocrit levels and platelet counts^13^. These increase the demand for reagents, specialized professionals, and laboratory structures. The emergence of COVID-19 contributed to overwhelming of laboratory services due to limited resources^4^. Therefore, despite testing of all suspected cases and contacts, as advised for COVID-19, in Espírito Santo State, many reported cases were evaluated exclusively using the clinicoepidemiological criteria due to the laboratory limitation for performing RT-PCR.

Primary healthcare, the entry-level to the health system for patients, is essential in epidemic situations. Professionals at this level must be well trained to manage co-occurring diseases with similar manifestations^4^ ^,^ ^7^. The emergence of COVID-19 in Espírito Santo State may have affected the management of patients with different conditions since the population was advised to seek for health services only for particular cases^5^. The impact of the lack of diagnosis and clinical follow-up of patients with different diseases on the outcome is unknown. Hospitalization may be required for arbovirus infections, but they were more likely for COVID-19, especially hospitalization to the intensive care unit. Deaths due to dengue, chikungunya, and COVID-19 were reported in the state, but additional deaths may be undetectable by the health system. In addition, coinfections may have occurred, but they were possibly undetected during this complex scenario.

Many infectious disease reports also affect epidemiological surveillance, increasing the efforts required to investigate and follow-up the reported cases^5^. Epidemiological surveillance service is also dependent on other services, such as the laboratory and outpatient care. Epidemiological surveillance is affected by delayed or absent laboratory results, underreporting of incident cases, or missing data in the case report form, compromising the capacity to capture the real epidemiological scenario and impairing appropriate epidemic responses. Therefore, underreporting of arbovirus infections and COVID-19 is likely in this situation^6^, and the epidemiological situation was probably far worse than that demonstrated by official data from Espírito Santo State.

This study presents some limitations inherent in ecological studies using secondary data, mainly because COVID-19 continues to affect the state’s health systems. Therefore, the number of reported cases may increase in the months after the study period due to the epidemiological service updates. This study evidences the impact of simultaneous epidemics that went beyond the socio-economic losses and was responsible for numerous deaths in Espírito Santo State, with many municipalities affected by at least two of concurrent epidemics, compromising the health system response. Additional studies should be performed to assess the effects of the COVID-19 pandemic in the state on the epidemiological profiles of non-communicable diseases, such as cardiovascular disease, cancer, chronic respiratory disease and diabetes, and communicable diseases, including dengue, chikungunya, and zika.

## SUPPLEMENTARY MATERIAL

**TABLE 1:** Dengue, Chikungunya, Zika and COVID-19 reports and incidence in the municipalities of Espírito Santo state, January to July 2020

Municipality	Dengue	Chikungunya	Zika	COVID-19	Population	Inc_Dengue	Inc_Chikungunya	Inc_Zika	Inc_COVID-19
AFONSO CLÁUDIO	32	7	20	541	30586	104,6	22,9	65,4	1768,8
ÁGUA DOCE DO NORTE	34	0	0	118	11019	308,6	0,0	0,0	1070,9
ÁGUIA BRANCA	0	0	0	213	9642	0,0	0,0	0,0	2209,1
ALEGRE	30	20	2	258	30084	99,7	66,5	6,6	857,6
ALFREDO CHAVES	72	3	10	267	14601	493,1	20,5	68,5	1828,6
ALTO RIO NOVO	0	0	0	120	7836	0,0	0,0	0,0	1531,4
ANCHIETA	28	2	0	601	29263	95,7	6,8	0,0	2053,8
APIACA	0	1	0	95	7567	0,0	13,2	0,0	1255,5
ARACRUZ	572	6	6	2544	101220	565,1	5,9	5,9	2513,3
ATÍLIO VIVAQUA	6	10	0	222	11936	50,3	83,8	0,0	1859,9
BAIXO GUANDU	170	182	46	554	30998	548,4	587,1	148,4	1787,2
BARRA DE S. FRANCISCO	64	2	0	351	44650	143,3	4,5	0,0	786,1
BOA ESPERANÇA	1406	15	30	291	15037	9350,3	99,8	199,5	1935,2
BOM JESUS DO NORTE	0	1	0	223	9936	0,0	10,1	0,0	2244,4
BREJETUBA	2	0	0	332	12404	16,1	0,0	0,0	2676,6
CACHOEIRO DE ITAPEMIRIM	5532	1877	132	3917	208972	2647,2	898,2	63,2	1874,4
CARIACICA	4784	1250	418	9369	381285	1254,7	327,8	109,6	2457,2
CASTELO	28	18	2	917	37534	74,6	48,0	5,3	2443,1
COLATINA	762	8	4	4559	122499	622,0	6,5	3,3	3721,7
CONCEIÇÃO DA BARRA	210	1	0	203	31063	676,0	3,2	0,0	653,5
CONCEIÇÃO DO CASTELO	46	0	8	161	12723	361,5	0,0	62,9	1265,4
DIVINO SÃO LOURENÇO	0	0	0	106	4304	0,0	0,0	0,0	2462,8
DOMINGOS MARTINS	20	5	6	529	33850	59,1	14,8	17,7	1562,8
DORES DO RIO PRETO	0	0	0	56	6749	0,0	0,0	0,0	829,8
ECOPORANGA	1046	3	2	374	22923	4563,1	13,1	8,7	1631,5
FUNDÃO	18	2	4	510	21509	83,7	9,3	18,6	2371,1
GOVERNADOR LINDENBERG	4	1	0	138	12709	31,5	7,9	0,0	1085,8
GUAÇUI	0	0	4	258	30867	0,0	0,0	13,0	835,8
GUARAPARI	168	150	4	2273	124859	134,6	120,1	3,2	1820,5
IBATIBA	6	1	0	345	26082	23,0	3,8	0,0	1322,8
IBIRAÇU	28	4	8	432	12479	224,4	32,1	64,1	3461,8
IBITIRAMA	1	1	0	76	8889	11,2	11,2	0,0	855,0
ICONHA	24	1	2	334	13860	173,2	7,2	14,4	2409,8
IRUPI	2	2	0	171	13377	15,0	15,0	0,0	1278,3
ITAGUAÇU	62	3	2	91	14066	440,8	21,3	14,2	647,0
ITAPEMIRIM	96	38	0	762	34348	279,5	110,6	0,0	2218,5
ITARANA	0	1	2	90	10555	0,0	9,5	18,9	852,7
IUNA	120	1	0	498	29161	411,5	3,4	0,0	1707,8
JAGUARÉ	262	0	14	380	30477	859,7	0,0	45,9	1246,8
JERÔNIMO MONTEIRO	6	2	2	163	12192	49,2	16,4	16,4	1336,9
JOÃO NEIVA	40	1	0	330	16668	240,0	6,0	0,0	1979,8
LARANJA DA TERRA	2	2	0	68	10947	18,3	18,3	0,0	621,2
LINHARES	6714	1	6	4720	173555	3868,5	0,6	3,5	2719,6
MANTENÓPOLIS	110	2	2	168	15350	716,6	13,0	13,0	1094,5
MARATAIZES	52	19	2	1029	38499	135,1	49,4	5,2	2672,8
MARECHAL FLORIANO	4	1	0	616	16694	24,0	6,0	0,0	3689,9
MARILÄNDIA	34	4	2	311	12833	264,9	31,2	15,6	2423,4
MIMOSO DO SUL	4	3	2	594	26153	15,3	11,5	7,6	2271,2
MONTANHA	120	0	0	165	18833	637,2	0,0	0,0	876,1
MUCURICI	16	0	0	48	5524	289,6	0,0	0,0	868,9
MUNIZ FREIRE	2	6	0	136	17465	11,5	34,4	0,0	778,7
MUQUI	0	0	4	279	15449	0,0	0,0	25,9	1805,9
NOVA VENÉCIA	216	3	4	671	50110	431,1	6,0	8,0	1339,1
PANCAS	64	1	0	391	23184	276,1	4,3	0,0	1686,5
PEDRO CANÁRIO	280	0	0	310	26184	1069,4	0,0	0,0	1183,9
PINHEIROS	232	0	0	371	27047	857,8	0,0	0,0	1371,7
PIÚMA	8	0	2	411	21711	36,8	0,0	9,2	1893,0
PONTO BELO	14	0	0	47	7863	178,0	0,0	0,0	597,7
PRESIDENTE KENNEDY	6	5	2	633	11574	51,8	43,2	17,3	5469,2
RIO BANANAL	18	1	6	219	19141	94,0	5,2	31,3	1144,1
RIO NOVO DO SUL	6	3	2	285	11622	51,6	25,8	17,2	2452,2
SANTA LEOPOLDINA	6	0	2	176	12224	49,1	0,0	16,4	1439,8
SANTA MARIA DE JETIBÁ	36	0	2	385	40431	89,0	0,0	4,9	952,2
SANTA TERESA	52	1	0	401	23590	220,4	4,2	0,0	1699,9
SÃO DOMINGOS DO NORTE	4	0	2	205	8638	46,3	0,0	23,2	2373,2
SÃO GABRIEL DA PALHA	868	2	102	1048	37947	2287,4	5,3	268,8	2761,7
SÃO JOSÉ DO CALÇADO	2	0	0	244	10556	18,9	0,0	0,0	2311,5
SÃO MATEUS	586	5	10	1732	130611	448,7	3,8	7,7	1326,1
SÃO ROQUE DO CANAÃ	14	0	8	146	12415	112,8	0,0	64,4	1176,0
SERRA	8286	830	120	12215	517510	1601,1	160,4	23,2	2360,3
SOORETAMA	42	0	0	585	30070	139,7	0,0	0,0	1945,5
VARGEM ALTA	16	5	8	330	21402	74,8	23,4	37,4	1541,9
VENDA N. DO IMIGRANTE	12	0	0	639	25277	47,5	0,0	0,0	2528,0
VIANA	482	62	14	1485	78239	616,1	79,2	17,9	1898,0
VILA PAVÃO	14	2	0	52	9208	152,0	21,7	0,0	564,7
VILA VALÉRIO	128	12	6	309	14080	909,1	85,2	42,6	2194,6
VILA VELHA	5762	886	146	13965	493838	1166,8	179,4	29,6	2827,9
VITÓRIA	4722	2616	140	12322	362097	1304,1	722,5	38,7	3403,0
**ESPÍRITO SANTO**	**44614**	**8092**	**3138**	**91483**	**4018650**	**1110,2**	**201,4**	**78,1**	**2276,5**
